# Early antidepressant treatment response prediction in major depression using clinical and TPH2 DNA methylation features based on machine learning approaches

**DOI:** 10.1186/s12888-023-04791-z

**Published:** 2023-05-01

**Authors:** Bingwei Chen, Zhigang Jiao, Tian Shen, Ru Fan, Yuqi Chen, Zhi Xu

**Affiliations:** 1 Department of Epidemiology and Biostatistics, School of Public health, Southeast University, Nanjing, 210009 China; 2 Department of Psychosomatics and Psychiatry, School of Medicine, Zhongda Hospital, Southeast University, Nanjing, 210009 China; 3 Department of Occupational Health and Poisoning Control, Shanghai Municipal Center for Disease Control and Prevention, Shanghai, 200336 China

**Keywords:** Major depressive disorder, TPH2, CpGs, Early antidepressant treatment response, Machine learning

## Abstract

**Objective:**

To identify DNA methylation and clinical features, and to construct machine learning classifiers to assign the patients with major depressive disorder (MDD) into responders and non-responders after a 2-week treatment into responders and non-responders.

**Method:**

Han Chinese patients (291 in total) with MDD comprised the study population. Datasets contained demographic information, environment stress factors, and the methylation levels of 38 methylated sites of tryptophan hydroxylase 2 (TPH2) genes in peripheral blood samples. Recursive Feature Elimination (RFE) was employed to select features. Five classification algorithms (logistic regression, classification and regression trees, support vector machine, logitboost and random forests) were used to establish the models. Performance metrics (AUC, F-Measure, G-Mean, accuracy, sensitivity, specificity, positive predictive value and negative predictive value) were computed with 5-fold-cross-validation. Variable importance was evaluated by random forest algorithm.

**Result:**

RF with RFE outperformed the other models in our samples based on the demographic information and clinical features (AUC = 61.2%, 95%CI: 60.1-62.4%) / TPH2 CpGs features (AUC = 66.6%, 95%CI: 65.4-67.8%) / both clinical and TPH2 CpGs features (AUC = 72.9%, 95%CI: 71.8-74.0%).

**Conclusion:**

The effects of TPH2 on the early-stage antidepressant response were explored by machine learning algorithms. On the basis of the baseline depression severity and TPH2 CpG sites, machine learning approaches can enhance our ability to predict the early-stage antidepressant response. Some potentially important predictors (e.g., TPH2-10-60 (rs2129575), TPH2-2-163 (rs11178998), age of first onset, age) in early-stage treatment response could be utilized in future fundamental research, drug development and clinical practice.

**Supplementary Information:**

The online version contains supplementary material available at 10.1186/s12888-023-04791-z.

## Background

Depression is a heterogeneous syndrome and encompasses various concomitant symptoms with varying reactions to treatment. Depressive symptoms are currently assessed through mainly subjective self-reported measures such as questionnaires and interviews. Clinical decision-making and treatment selection depend primarily on the clinical experience and professional judgment of psychiatrists because of no biomarker available for diagnostic or prognostic testing [1]. Selective serotonin reuptake inhibitors (SSRIs) are the common first-line agents used to treat major depressive disorder (MDD) [2, 3], but over two-third of the patients who received SSRIs treatment failed to achieve symptom remission [4]. In response to antidepressants, it is estimated that up to 42% of the individual variation was explained by genetic factors. From this perspective, the genetic makeup of patients may help to select an appropriate medication for different individuals and allow adjustment of drug dosage according to the likelihood of optimal therapeutic effect with least side effects. However, no significant discovery of specific genetic polymorphisms have been reported [5].

Other studies have demonstrated a link between epigenetic modifications and MDD, including methylation [6]. DNA methylation occurs at the cytosine pyrimidine ring of cytosine–phosphate–guanine dinucleotide sites (CpGs), which are particularly common in the promoter regions [7]. DNA methylation in combination with genetic predisposition and environmental exposure could serve as a prognostic factor of disease occurrence or personal risk [8]. Blood methylation patterns have also been evidenced to be associated with the risk of long-term depression [9]. Variation in the TPH2 gene was explored as a possible factor since genetic variability related to the brain serotonin system has a significant impact on MDD [10]. Our previous studies indicate that TPH2 single nucleotide polymorphisms (SNPs) (rs7305115, the haplotype of rs7305115 and rs4290270 [11], rs1487278, and rs2171363 compounding childhood adversity [12]) are closely associated with the antidepressant response in Chinese MDD patients. Equally important, the investigation on potential biomarkers for predicting response to depression treatment is urgently need due to the high rate of treatment resistance, the increased suicide rate in non-reactive MDD patients, and the crushing economic burden. Predicting patient’s response to an early-stage treatment can help clinicians to optimize therapeutic methods at an earlier stage, which will reduce morbidity and improve patients’ life quality. To the best of our knowledge, only little research has previously been undertaken to predict early responders in MDD based on DNA methylation.

Machine learning (ML) has been used as a valuable tool to assist clinicians to make more thoughtful decisions for their patients due to its capability to manage complex and voluminous datasets with various types of clinical and genomic data [13]. Machine learning has also emerged as a powerful tool to uncover unknown features from large-scale epigenetic data [14]. MDD classification and prediction, based on machine learning and neuroimaging information (e.g., MRI data), have been investigated [15] and systematically summarized [16]. An ensemble learning model has been reported to integrate imaging and genetic information for individualized baseline prediction of response to a 2-week antidepressant treatment in 98 MDD inpatients [17]. Along with clinical and genetic factors assessed at baseline, some machine learning models could generate predictors for treatment response assessed at Week 5 [18]. Deep learning models were developed to evaluate antidepressant treatment outcomes in Taiwanese subjects [19].

Therefore, we sought to identify DNA methylation features and to construct supervised machine learning classifiers to assign responders and non-responders after a 2-week treatment. These potential predictors may enhance the understanding of the epigenetic mechanisms of early prognosis in MDD.

## Materials and methods

Standards and guidelines of machine learning in psychiatry were followed when this study was conducted and reported [20].

### Participants and clinical assessments

This study included 291 inpatients in a tertiary hospital who were diagnosed as major depressive disorders. Patient eligibility was determined based on the criteria of the Diagnostic and Statistical Manual of the American Psychiatric Association, Fourth Edition (DSM-IV). Blood samples were collected before antidepressant treatment.

All patients met the following criteria: Han Chinese, 18–65 years old, baseline 17-item Hamilton Depression Rating Scale (HAMD-17) [21] scores > 17 points, and their depressive symptoms lasted at least 2 weeks. All patients had just been diagnosed or had recently relapsed and had not been on medication for at least two weeks prior to enrollment. All diagnoses were made independently by two psychiatrists with professional tenure or higher, and confirmed by a third psychiatrist. Participants had never been diagnosed with other DSM-IV Axis I diagnosis (including substance use disorder, schizophrenia, affective disorder, bipolar disorder, generalized anxiety disorder, panic disorder, obsessive-compulsive disorder). They had never been diagnosed with personality disorder or mental retardation. Patients with a history of organic brain syndrome, endocrine, and primary organic diseases, or other medical conditions that would hinder psychiatric evaluation were excluded from the study. Other exclusion criteria included blood, heart, liver, and kidney disorders; electroconvulsive therapy in the past 6 months; or an episode of mania in the previous 12 months. Pregnant and nursing females were also excluded from participation.

All study subjects in the study endorsed written consent that was approved by the Zhongda Hospital Ethics Committee (2016ZDSYLL100-P01) under the Declaration of Helsinki.

### Demographic and clinical data

Response was defined as ≥ 50% reduction in HAMD-17 scores from baseline to two weeks [22]. Accordingly, the two-week treatment participants were divided into two groups, responders and non-responders.

Two retrospective self-report questionnaires, the Childhood Trauma Questionnaire (28-item short-form, CTQ-SF) and the Life Events Scale (LES), were used to evaluate recent stress exposures and childhood adversities, respectively. The evaluation of LES and CTQ scales was completed by the same nurse using consistent, scripted language. LES is a self-assessed questionnaire composed of 48 items, reflecting both positive and negative life events experienced within the past year. The LES is divided into positive and negative life events (NLES). The CTQ-SF was dichotomized for use in the gene-environment interaction analyses.

Twelve considered demographic and clinical features are age, gender, years of education, marital status, family history, first occurrence or not, age of onset, number of occurrences, illness duration, HAMD-17, NLES and CTQ-SF baseline scores (Supplemental Material Table 1).

### Genetic information

Primers were previously designed by us to encompass 100 bp upstream and 100 bp downstream of TPH2 SNPs that showed a significant association with the antidepressant response, as well as with GC sequence content of CpGs > 20% after methylation [11, 12]. Out of the total 24 TPH2 SNPs, only 11 SNPs (rs7305115, rs2129575, rs11179002, rs11178998, rs7954758, rs1386494, rs1487278, rs17110563, rs34115267, rs10784941, rs17110489) met the DNA methylation status criteria of the sequences to be detected (Supplemental Material Table 2). Methylation levels of 38 TPH2 CpGs were calculated and presented as the ratio of the number of methylated cytosines to the total number of cytosines.

### Missing value handling

**Fig. 1:**
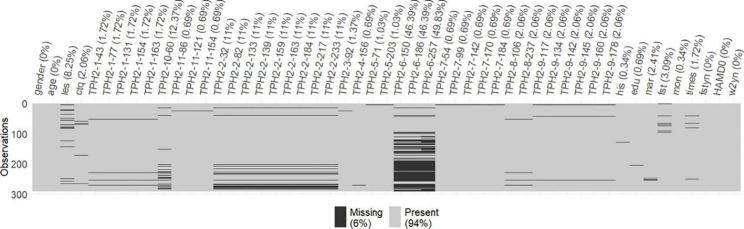
Missingness pattern in the DNA methylation data set

In the data set comprising 291 observations of 51 variables (12 demographic and clinical features, 38 CpGs’ methylation levels and 1 response variable), 6% entries were missing (see Fig. 1). Of the CpGs’ methylation levels, 3 CpGs (TPH2-7-99, TPH2-7-142, TPH2-7-170) were excluded because they had more than 45% missing values. Due to the randomness of experimental/technological errors and interrelatedness of the variables, missing completely at random (MCAR)/missing at random (MAR) was assumed for the DNA methylation data and the mean imputation can deal with the missing data [23, 24]. The values of other features with missing values were imputed with mode and mean in the case of categorical and numerical features, respectively.

### Classification modeling using machine learning algorithms

Normalization (Linear transformation) was used to improve the numerical stability of the model and reduce training time [25]. To avoid overfitting when harnessing maximum amount of data, cross-validation (CV) using entire sample was used to report prediction performance. The CV was 5-fold and the averaged prediction metrics including the area under the receiver operating curve (AUC), F-Measure, G-Mean, accuracy, sensitivity, specificity, positive predictive value (PPV) and negative predictive value (NPV) were reported. Hyperparameter tuning was based on AUC with random search using the caret default tuning settings. A packaging method (Recursive Feature Elimination with random forest, RFE-RF) [26] with 5-fold CV was employed to select the features that contributed the most to the prediction of the early antidepressant response in MDD patients. The variable importance was also estimated using random forest. For better replicability, the 5-fold CV procedure was repeated 10 times.

ML methods were implemented via their interface with the open-source R package “caret” in a standardized and reproducible way. Five different supervised ML algorithms were used in this study, including logistic regression, classification and regression trees (CART), support vector machine with radial basis function kernel (SVM-RBF), a boosting method (logitboost) and random forests (RF) to develop predictive models. All analyses were implemented in R statistical software (version 4.0.4). We utilized the caret package which implements rpart, caTools, e1071 and RandomForest packages for CART, logitboost, SVM-RBF and RF, respectively.

## Results

### Demographic and clinical characteristics of patients

After a two-week antidepressant therapy, 180 (61.9%) of MDD patients met the criteria for responding to antidepressants. 35.1% (n = 102) of patients were males and the mean age was 46.4, 46.7% (n = 136) of patients were first occurrence and the mean of baseline HAMD-17 was 22.95. Further details of the baseline characteristics of demographic and clinical features are shown in Table 1.

Prior to treatment, no statistically significant differences in the demographic features were observed between the responders and non-responders. The demographic features include age, years of education, family history, education duration, marital status, age of onset, illness duration, NLES and CTQ scores. However, the predominant proportion of women versus men was significant (P = 0.011). Moreover, the HAMD-17 baseline scores of responders were significantly lower than those of non-responders (P = 0.030).

**Table 1:** Demographic and clinical features at baseline

Variables	Responders (N = 180)	Non-responders (N = 111)	Total	P value
Gender, male (n (%))	53 (29.4)	49 (44.1)	102 (35.1)	0.011
Age (mean (SD))	46.94 (13.19)	45.63 (14.25)	46.40 (13.65)	0.335
NLES (median [IQR])	16.00 [5.75, 32.00]	12.00 [3.00, 37.00]	13.00 [4.00, 32.00]	0.558
CTQ (mean (SD))	47.58 (9.11)	48.50 (8.56)	47.93 (8.90)	0.399
Family history, yes (n (%))	33 (18.4)	19 (17.1)	52 (17.9)	0.776
Years of education (mean (SD))	10.80 (3.68)	10.51 (4.34)	10.69 (3.94)	0.537
Marital status, yes (n (%))	150 (84.7)	85 (79.4)	235 (80.8)	0.325
First occurrence or not, yes (n (%))	88 (48.9)	48 (43.2)	136 (46.7)	0.414
Age of onset (mean (SD))	42.09 (13.25)	39.84 (14.54)	41.24 (13.77)	0.185
Illness duration (median [IQR])	24.00 [6.00, 72.00]	24.00 [11.50, 84.00]	24.00 [6.00, 72.00]	0.268
Number of occurrences (mean (SD))	1.89 (1.29)	2.24 (1.68)	2.02 (1.46)	0.046
Baseline HAMD-17 (mean (SD))	22.55 (3.86)	23.59 (4.06)	22.95 (3.97)	0.030

### Model performance

#### Clinical-based classification

**Fig. 2:**
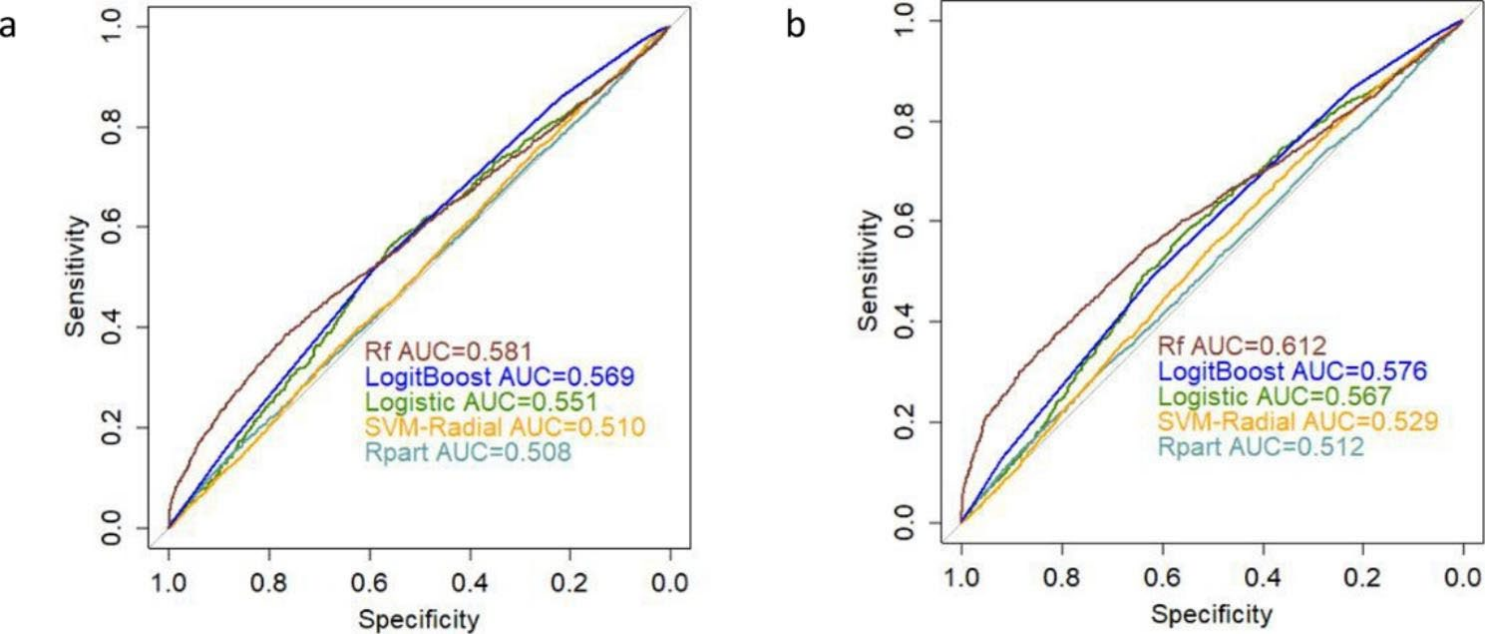
ROC curves of different classifiers based on clinical data **a**) ROC curves without RFE. **b**) ROC curves with RFE.

Formulas in classifiers were summarized in Table 2. The results illustrated in Table 3 were obtained when we employed depression severity scores (HAMD-17), together with demographic and clinical features, without / with performing feature selection. A comparison of the classifiers’ performances led to the conclusion that RF reached the highest averaged AUC, and RFE improved the AUC from 58.1% (95%CI: 56.9-59.3%) to 61.2% (95%CI: 60.1-62.4%), but without statistically significant difference (P = 0.067). ROC curves of different classifiers based on demographic and clinical features were demonstrated in Fig. 2. It can also be concluded by comparing the other values that SVM-RBF had highest F-measure and sensitivity values and that logitboost exhibited a higher G-mean, accuracy and specificity than the rest of the algorithms.

**Table 2:** Formulas in classifiers

Data set	Feature selection	Formulas	Number
Only demographic and clinical features	Without RFE	w2yn **~** gen + age + les + ctq + his + edu + mar + fst + mon + times + fstyn + HAMD0	12
	With RFE	w2yn **~** HAMD0 + age + times + les + ctq + gen + edu + fst + fstyn + mar	10
Only TPH2 CpGs	Without RFE	w2yn **~** t1 + t2 + … + t38^a^	35
	With RFE	w2yn **~** t6 + t12 + t13 + t11 + t29 + t17 + t15 + t32 + t33 + t16 + t2 + t18 + t14 + t28 + t20 + t31	16
Both	Without RFE	w2yn **~** gen + age + les + ctq + his + edu + mar + fst + mon + times + fstyn + HAMD0 + t1 + t2 + … + t38 ^a^	47
	With RFE	w2yn **~** t12 + t17 + t6 + t15 + t13 + t32 + gen + t11 + times + age + t14 + t29 + HAMD0 + t16 + fst + t18 + mar + les + t31 + t33 + ctq + t4 + t2	23

^a^ 35 CpGs features were included in the model, and 3 CpGs (TPH2-7-99、TPH2-7-142、TPH2-7-170) were excluded with more than 45% missing values

**Table 3:** Averaged prediction metrics for each classifier based on clinical characteristics

Feature selection	Classifier	ROC (95%CI)	F-Measure	G-Mean	Accuracy	Sensitivity	Specificity	PPV	NPV
Without RFE	Logistic	0.551 (0.529–0.572)	0.710	0.428	0.589	0.813	0.225	0.630	0.426
	Rpart	0.508 (0.496–0.521)	0.684	0.422	0.563	0.766	0.233	0.618	0.380
	SVM-RBF	0.510 (0.498–0.523)	0.762	0.113	0.617	0.990	0.013	0.619	0.445
	LogitBoost	0.569 (0.557–0.580)	0.724	0.523	0.624	0.786	0.348	0.662	0.501
	RF	0.581 (0.569–0.593)	0.687	0.471	0.577	0.750	0.296	0.633	0.422
With RFE	Logistic	0.567 (0.545–0.588)	0.722	0.444	0.604	0.830	0.237	0.638	0.462
	Rpart	0.512 (0.500-0.525)	0.685	0.428	0.565	0.766	0.239	0.620	0.386
	SVM-RBF	0.529 (0.516–0.541)	0.758	0.177	0.615	0.975	0.032	0.620	0.441
	LogitBoost	0.576 (0.564–0.587)	0.726	0.539	0.629	0.782	0.372	0.669	0.513
	RF	0.612 (0.601–0.624)	0.694	0.487	0.587	0.756	0.314	0.641	0.442

#### CpGs-Based classification

**Fig. 3:**
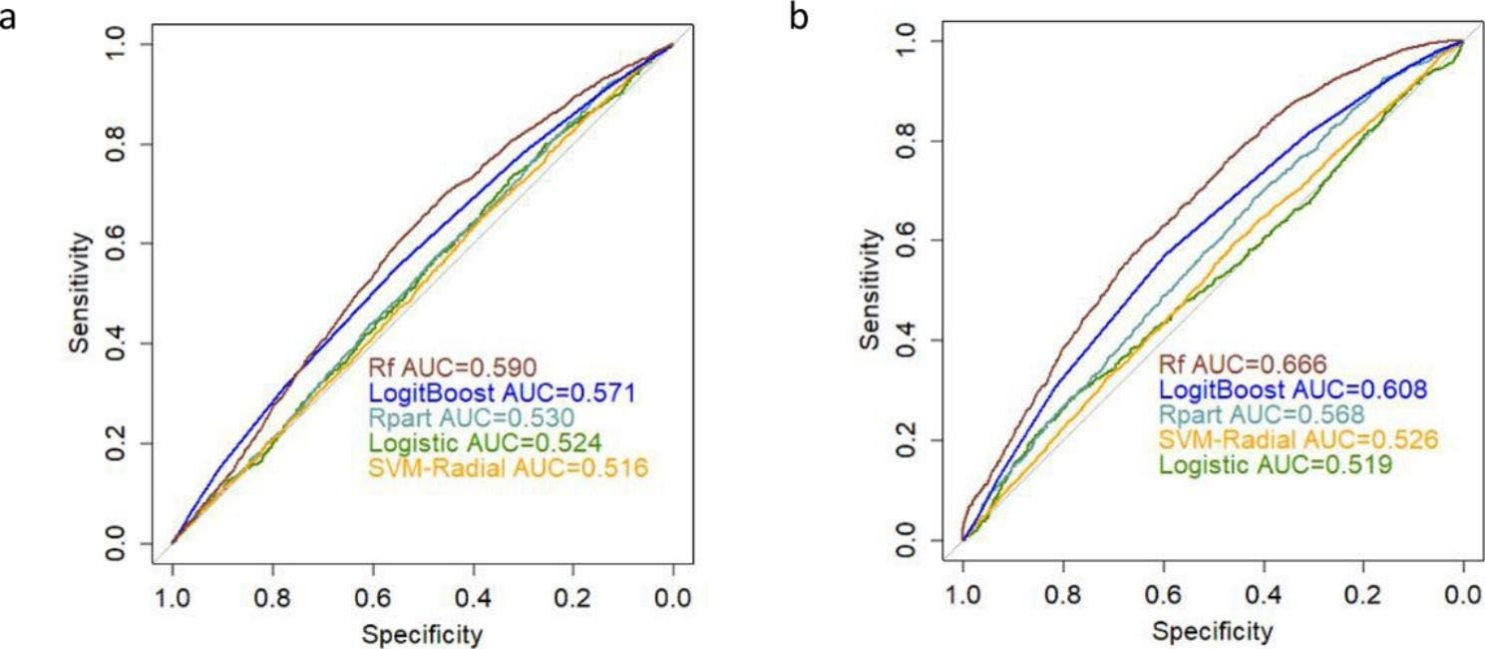
ROC curves of different classifiers based on CpGs data **a**) ROC curves without RFE. **b**) ROC curves with RFE.

We then examined the discriminant methylation potential of 38 TPH2 CpGs. Formulas in classifiers were listed in Table 2. The results that we achieved when CpG sites were used for classification without/with RFE were summarized in Table 4. It can be concluded that the best performing classification algorithm is RF. Additionally, RFE improved the AUC from 59.0% (95%CI: 57.8-60.3%) to 66.6% (95%CI: 65.4-67.8%) and accuracy from 62.8 to 67.0%, with significant differences (P = 0.004). ROC curves of different classifiers based on TPH2 CpGs data were shown in Fig. 3. SVM-RBF obtained F-measure above 75% and sensitivity above 98%.

**Table 4:** Averaged prediction metrics for each classifier based on TPH2 CpGs

Feature selection	Classifier	ROC (95%CI)	F-Measure	G-Mean	Accuracy	Sensitivity	Specificity	PPV	NPV
Without RFE	Logistic	0.524 (0.502–0.546)	0.661	0.432	0.544	0.720	0.259	0.612	0.363
	Rpart	0.530 (0.517–0.542)	0.677	0.472	0.569	0.731	0.305	0.630	0.411
	SVM-RBF	0.516 (0.503–0.528)	0.762	0.045	0.615	0.994	0.002	0.618	0.171
	LogitBoost	0.571 (0.559–0.583)	0.691	0.536	0.599	0.717	0.401	0.660	0.466
	RF	0.590 (0.578–0.603)	0.750	0.407	0.628	0.901	0.184	0.642	0.534
With RFE	Logistic	0.519 (0.498–0.540)	0.710	0.378	0.580	0.831	0.172	0.619	0.386
	Rpart	0.568 (0.556–0.581)	0.683	0.522	0.588	0.716	0.381	0.652	0.453
	SVM-RBF	0.526 (0.514–0.539)	0.760	0.160	0.617	0.981	0.026	0.620	0.458
	LogitBoost	0.608 (0.596–0.620)	0.725	0.574	0.638	0.756	0.436	0.685	0.524
	RF	0.666 (0.654–0.678)	0.763	0.558	0.670	0.859	0.363	0.686	0.614

#### Clinical-and-CpGs-Based classification

**Fig. 4:**
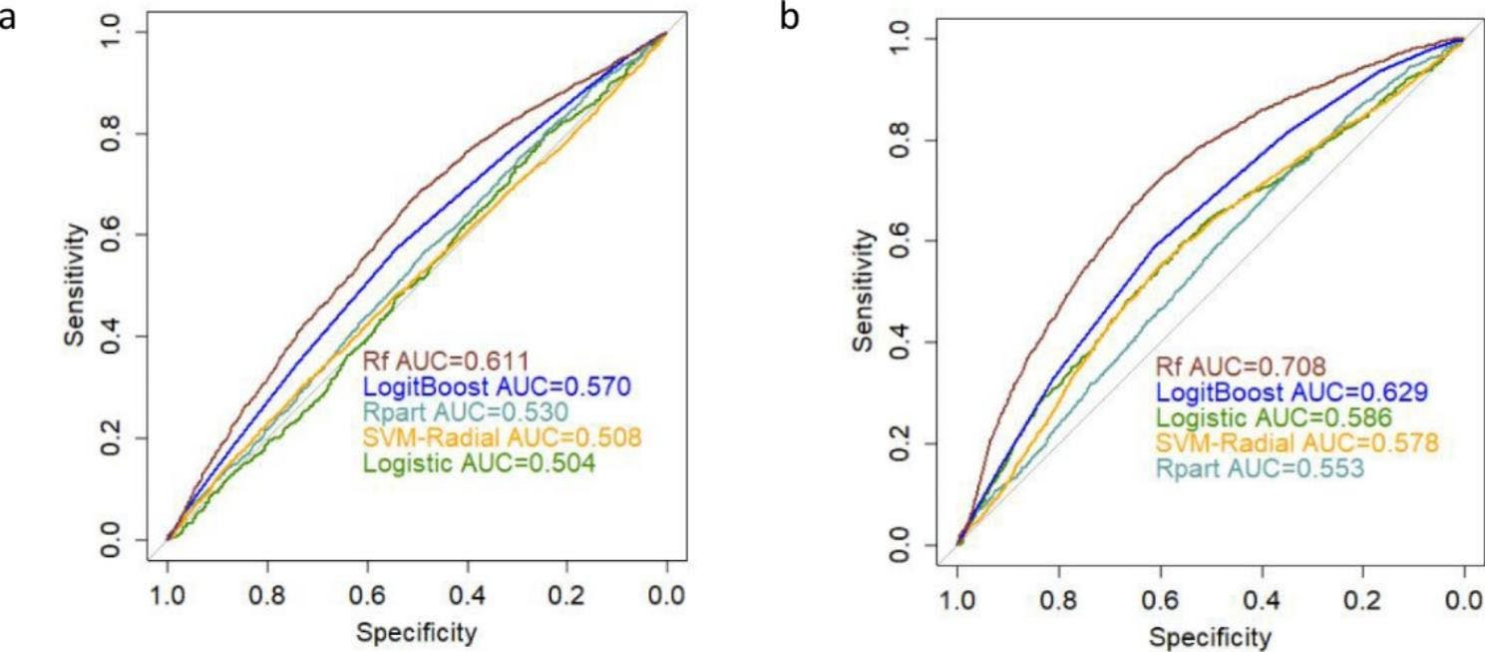
ROC curves of different classifiers based on clinical and CpGs data **a**) ROC curves without RFE. **b**) ROC curves with RFE.

The results that were gathered from the clinical and TPH2 CpGs data, without/with performing feature selection, were summarized in Table 5, which suggest that RF clearly outperformed the other four classifiers. Additionally, RFE improved the AUC from 61.1% (95%CI: 59.9-62.3%) to 72.9% (95%CI: 71.8-74.0%) and accuracy from 62.5 to 70.0%, the differences were significant (P < 0.001). ROC curves of different classifiers based on clinical and TPH2 CpGs data were illustrated in Fig. 4, suggesting that SVM-RBF obtained F-measure above 75% and sensitivity above 91%.

RF was established as the optimal performing model (72.9% AUC, 78.5% F-Measure, 59.8% G-Mean, 70.0% accuracy, 88.2% sensitivity, 0.706 PPV and 0.679 NPV), with 23 features selected by RFE. RF was thus used to estimate the variable importance of these 23 features for classification. The top-15 variables and variable importance were shown in Table 3 of Supplemental Material. Top-10 variables are TPH2-10-60 (rs2129575), TPH2-2-163 (rs11178998), TPH2-7-170 (rs34115267), TPH2-8-237 (rs10784941), TPH2-1-77 (rs7305115), TPH2-2-133 (rs11178998), TPH2-2-139 (rs11178998), TPH2-8-106 (rs10784941), age of first onset and TPH2-2-159 (rs11178998).

**Table 5:** Averaged prediction metrics for each classifier based on clinical characteristics and TPH2 CpGs

Feature selection	Classifier	ROC (95%CI)	F-Measure	G-Mean	Accuracy	Sensitivity	Specificity	PPV	NPV
Without RFE	Logistic	0.504 (0.482–0.525)	0.660	0.472	0.554	0.700	0.318	0.625	0.395
	Rpart	0.530 (0.518–0.543)	0.669	0.486	0.566	0.710	0.332	0.633	0.414
	SVM-RBF	0.508 (0.496–0.521)	0.759	0.094	0.613	0.985	0.009	0.617	0.270
	LogitBoost	0.570 (0.558–0.582)	0.690	0.537	0.598	0.712	0.405	0.660	0.464
	RF	0.611 (0.599–0.623)	0.744	0.431	0.625	0.880	0.211	0.644	0.520
With RFE	Logistic	0.590 (0.569–0.611)	0.707	0.446	0.589	0.799	0.249	0.633	0.433
	Rpart	0.559 (0.547–0.572)	0.708	0.506	0.605	0.775	0.331	0.653	0.476
	SVM-RBF	0.630 (0.625–0.635)	0.753	0.378	0.627	0.918	0.156	0.638	0.540
	LogitBoost	0.617 (0.612–0.623)	0.717	0.583	0.635	0.742	0.458	0.689	0.523
	RF	0.729 (0.718–0.740)	0.785	0.598	0.700	0.882	0.405	0.706	0.679

## Discussion

Given the phenotypic complexity of the antidepressant response, clinical data are insufficient to guide the treatment selection for each patient [27]. Methylation marks are potential biomarkers reflecting variation within the central nervous system [28] and they are very stable in bio-samples. TPH2 contributes to altered neuronal serotonin (5-HT) function, which are associated with MDD or suicidal behavior. Methylation of CpGs in the TPH2 promoter area affects gene expression [29]. TPH2 is therefore considered as a candidate gene for MDD and the pharmacogenetics of the antidepressant reaction [30]. However, the precise mechanism of the serotonin system in MDD remains to be clarified [31, 32]. In the present study, clinical features and information on TPH2 methylation were gathered to establish prediction models for the early-stage antidepressant response in the Chinese Han population, leading to the identification of important CpGs as potential biomarkers in the early MDD treatment.

The response rate (a 50% reduction of HAMD-17 scores at 2 weeks relative to baseline) is 61.9% in our study. All patients were treated with a single antidepressant appropriate to the clinical indication. Specifically, the antidepressants include Selective Serotonin Reuptake Inhibitors (SSRI) (n = 177) and non-SSRIs (n = 114). The non-SSRIs include serotonin and nor-epinephrine reuptake inhibitors (SNRI) (n = 94), noradrenergic and selective serotonergic antidepressants (NaSSA(s) (n = 13), and serotonin antagonists and reuptake inhibitors (SARI) (n = 7). Antipsychotics and mood stabilizers were not used for concomitant treatment, except for a low dose of benzodiazepine anxiolytic for the treatment of insomnia in some cases. In contrast, the response rate reported in the literature is 36.5% for the MDD patients treated with fluoxetine [33]. Our response rate is indeed higher than that described in the literature. The relatively higher response rate in our study is probably caused by: (1) samples that we collected from in-patients who might have received better care and support from clinic staff at the hospital; (2) the baseline HAMD-17 of in-patients was 22.95 in this study, indicating that the symptoms of these in-patients were mild and not refractory; and (3) the patients were discharged at 2 weeks. They might expect to be discharged, which could cause a bias in the evaluation at 2 weeks. The treatment rebound may occur in patients after discharge from the hospital. Lacking follow up is the limitation of this study. This study more focuses on the short-term effect in 2 weeks, from the perspective of machine learning to analyses its impact factors. Additionally, antidepressant drug dosage was adjusted as needed during the study.

Our findings suggest the better prediction performance of the models with the selected features. The prediction model can synergize clinical data with DNA methylation data to improve the prediction power of our classifiers and clinical prognostic evaluation [34]. RF with RFE in this study outperformed the other reported models according to the clinical features (AUC = 61.2%, 95%CI: 60.1-62.4%) / TPH2 CpGs features (AUC = 66.6%, 95%CI: 65.4-67.8%) / both clinical and TPH2 CpGs features (AUC = 72.9%, 95%CI: 71.8-74.0%). Compared with the other three evaluated models, logistic and rpart classifier have the relatively lower values of ROC-AUC and are less accurate. The sensitivity and specificity were associated with optimal threshold. For example, the classifier’s Youden index gets maximum value at a threshold of 0.581, the sensitivity and specificity of RF with RFE based on clinical and TPH2 CpGs features are 0.882 and 0.405. If we reduce threshold, the sensitivity will decrease and the specificity will increase. In our study, the specificities of all models are not high, which means the classifiers are better at identifying likely responders than at identifying likely non-responders. We also chose AUC to represent the performance of models because AUC combines sensitivity and specificity under different thresholds and it is more often to evaluate for model performance.

Encouraged by the good performance of the RF model, we further investigated the contribution from TPH2 methylation. RFE helped us to identify a small subset of discriminative and predictive CpGs in SNP TPH2 sites. These SNP sites with high importance values have been demonstrated to have a connection with neuropsychiatric conditions, and our research results are consistent with those reported data. A recent research has shown that rs2129575 might be a susceptibility gene underlying heroin addiction [35]. A meta-analysis of the studies in BD showed that the fixed summary OR for rs11178998 (1184 cases and 1585 controls) was 1.33 (95% CI: 1.09–1.61) [36]. Another systematic review and meta-analysis showed that TPH2 SNPs rs11178998 is associated with one or more psychopathological conditions [37]. Also, a significant association with schizophrenia for rs10784941 (p = 0.009, OR minor G-allele 0.82 [0.71–0.95]) was observed in the discovery sample consisting of 788 schizophrenia patients and 688 controls [38]. Moreover, meta-analysis results based on nine SNPs in the TPH2 gene revealed that rs7305115 was associated with suicidal behavior under a fixed effect model [39]. Additionally, rs34115267 was selected into its cohort study due to its risk patterns and processes for psychopathology emerging in adolescence (ROOTS) project [40]. Collectively, in this study, the good performance of ML implied its potential to predict MDD and to assist clinicians in making more objective and efficient decisions. Even though we cannot advocate the applicability of these clinical prediction models at this moment, our study provided the probability of exploring more blood-based biomarkers for MDD prediction, serving as a step towards precision medicine in psychiatry. These predictive SNPs in early-stage treatment response could be utilized in many contexts, such as fundamental research, drug development and clinical practice.

Besides, demographic and clinical variables (e.g., age of first onset, age) were considered important features for early-stage treatment response prediction. Further evidence was provided that age and gender moderate response to antidepressants [41]. In actual statistical analyses, most research on MDD included age and gender as key covariates [42, 43].

However, the following limitations should be considered when interpreting the findings of this study. In view of the relatively small sample size, 5-fold CV was employed to avoid overfitting of the models, and future prospective studies utilizing larger sample sizes are warranted to confirm our findings. Also, the following predictors could be selected to reduce the uncertainty associated with the prediction of the early-stage antidepressant response: electroencephalogram (EEG) [44] and environmental factors [45], such as diet, alcohol consumption, stress and smoking status.

## Conclusion

In conclusion, the effects of TPH2 on the early-stage antidepressant response was explored by supervised machine learning algorithms. On the basis of the baseline depression severity and the TPH2 CpG sites, machine learning approaches can enhance our ability to predict the early-stage antidepressant response. Some potentially important predictors (e.g., TPH2-10-60(rs2129575), TPH2-2-163(rs11178998), age of first onset, age) in early-stage treatment response could be utilized in future fundamental research, drug development and clinical practice.

## Electronic supplementary material

Below is the link to the electronic supplementary material.

Supplementary Material 1 Table 1

Supplementary Material 2 Table 2

Supplementary Material 3 Table S3

## Data Availability

The datasets generated and/or analyzed during the current study are not publicly available due to the sensitive nature of the raw data but are available from the corresponding author on reasonable request.

## Abbreviations

MDD: Major depressive disorder
TPH2: Tryptophan hydroxylase 2
RFE: Recursive feature elimination
AUC: The area under the receiver operating curve
PPV: Positive predictive value
NPV: Negative predictive value
CpGs: Cytosine–phosphate–guanine dinucleotide sites
SSRIs: Selective serotonin reuptake inhibitors
SNPs: Single nucleotide polymorphisms
ML: Machine learning
DSM-IV: Diagnostic and Statistical Manual of the American Psychiatric Association, Fourth Edition
HAMD-17: 17-item Hamilton Depression Rating Scale
CTQ-SF: The Childhood Trauma Questionnaire (28-item short-form)
LES: The life vents scale
NLES: Negative life events
MCAR: Missing completely at random
MAR: Missing at random
CV: Cross-validation
RFE-RF: Recursive feature elimination with random forest
CART: Classification and regression trees
SVM-RBF: Support vector machine with radial basis function kernel
RF: Random forests
VI: Variable importance
