# Supplementary material for: Early antidepressant treatment response prediction in major depression using clinical and TPH2 DNA methylation features based on machine learning approaches
Source: BMC Psychiatry. 2023 May 1;23:299. doi: 10.1186/s12888-023-04791-z (PMC10150459; doi:10.1186/s12888-023-04791-z)
Supplement: Supplementary file 1 — Supplementary Material 1 Table 1 [file 12888_2023_4791_MOESM1_ESM.docx]

**Supplementary Material Table 1** Code sheet for the features

| Features | Factors | Values |
| --- | --- | --- |
| gen | Gender | 1-male, 2-female |
| age | Age | years |
| les | Negative life events score | scores |
| ctq | CTQ score | scores |
| his | Family history | 0-no, 1-yes |
| edu | Years of education | years |
| mar | Married or not | 0-no, 1-yes |
| fst | Age of first onset | years |
| mon | Total disease duration | months |
| times | Incidence | times |
| fstyn | First occurrence or not | 0-no, 1-yes |
| t1-t38 | DNA methylation levels of 38 TPH2 gene loci | % |
| hd0 | HDRS score at entry | scores |
| hd2 | HDRS score after two-week treatment | scores |
| w2yn | Treatment response | 0-non-response, 1-response |

Note: HDRS includes 17 questions. Normal: a total score of <7, Possible depression: a total score of 7 to 17, Depression: a total score of 17 to 24, Severe depression: a total score of> 24.

Abbreviation: CTQ, childhood trauma questionnaire; HDRS, hamilton depression scale
