# Supplementary material for: Early antidepressant treatment response prediction in major depression using clinical and TPH2 DNA methylation features based on machine learning approaches
Source: BMC Psychiatry. 2023 May 1;23:299. doi: 10.1186/s12888-023-04791-z (PMC10150459; doi:10.1186/s12888-023-04791-z)
Supplement: Supplementary file 2 — Supplementary Material 2 Table 2 [file 12888_2023_4791_MOESM2_ESM.docx]

**Supplementary Material Table 2** Features of 38 methylation sites and corresponding SNP sites

| Features | Methylated site | SNP site | Features | Methylated site | SNP site |
| --- | --- | --- | --- | --- | --- |
| t1 | TPH2-1-43 | rs7305115 | t20 | TPH2-4-156 | rs1386494 |
| t2 | TPH2-1-77 | rs7305115 | t21 | TPH2-5-71 | rs1487278 |
| t3 | TPH2-1-131 | rs7305115 | t22 | TPH2-5-203 | rs1487278 |
| t4 | TPH2-1-154 | rs7305115 | t23 | TPH2-6-150 | rs17110563 |
| t5 | TPH2-1-163 | rs7305115 | t24 | TPH2-6-186 | rs17110563 |
| t6 | TPH2-10-60 | rs2129575 | t25 | TPH2-6-257 | rs17110563 |
| t7 | TPH2-11-86 | rs11179002 | t26 | TPH2-7-54 | rs34115267 |
| t8 | TPH2-11-121 | rs11179002 | t27 | TPH2-7-99 | rs34115267 |
| t9 | TPH2-11-154 | rs11179002 | t28 | TPH2-7-142 | rs34115267 |
| t10 | TPH2-2-32 | rs11178998 | t29 | TPH2-7-170 | rs34115267 |
| t11 | TPH2-2-82 | rs11178998 | t30 | TPH2-7-184 | rs34115267 |
| t12 | TPH2-2-133 | rs11178998 | t31 | TPH2-8-106 | rs10784941 |
| t13 | TPH2-2-139 | rs11178998 | t32 | TPH2-8-237 | rs10784941 |
| t14 | TPH2-2-159 | rs11178998 | t33 | TPH2-9-117 | rs17110489 |
| t15 | TPH2-2-163 | rs11178998 | t34 | TPH2-9-134 | rs17110489 |
| t16 | TPH2-2-184 | rs11178998 | t35 | TPH2-9-142 | rs17110489 |
| t17 | TPH2-2-217 | rs11178998 | t36 | TPH2-9-145 | rs17110489 |
| t18 | TPH2-2-233 | rs11178998 | t37 | TPH2-9-160 | rs17110489 |
| t19 | TPH2-3-92 | rs7954758 | t38 | TPH2-9-178 | rs17110489 |
