# Supplementary material for: Early antidepressant treatment response prediction in major depression using clinical and TPH2 DNA methylation features based on machine learning approaches
Source: BMC Psychiatry. 2023 May 1;23:299. doi: 10.1186/s12888-023-04791-z (PMC10150459; doi:10.1186/s12888-023-04791-z)
Supplement: Supplementary file 3 — Supplementary Material 3 Table S3 [file 12888_2023_4791_MOESM3_ESM.docx]

**Supplementary Material Table S3** Variable importance of top-15 variables

| Variable | SNP site | VI |
| --- | --- | --- |
| TPH2-10-60 | rs2129575 | 8.1541 |
| TPH2-2-163 | rs11178998 | 7.9666 |
| TPH2-7-170 | rs34115267 | 7.7467 |
| TPH2-8-237 | rs10784941 | 7.6124 |
| TPH2-1-77 | rs7305115 | 7.5952 |
| TPH2-2-133 | rs11178998 | 6.9398 |
| TPH2-2-139 | rs11178998 | 6.5314 |
| TPH2-8-106 | rs10784941 | 6.3994 |
| fst |  | 6.3992 |
| TPH2-2-159 | rs11178998 | 6.3923 |
| age |  | 6.2686 |
| TPH2-1-154 | rs7305115 | 6.1999 |
| ctq |  | 6.0870 |
| TPH2-9-117 | rs17110489 | 6.0063 |
| TPH2-2-82 | rs11178998 | 5.9545 |

Abbreviations: VI: Variable importance; fst: Age of first onset; CTQ: Childhood trauma questionnaire
